# Incidence and healthcare costs of symptomatic dengue in an urban, city-wide cohort in South India

**DOI:** 10.1186/s12879-026-12545-x

**Published:** 2026-01-23

**Authors:** Nikhil Sahai, Nimi Elizabeth Thomas, Dilesh Kumar, Hannah Miraculine, Joshua Anish Selwyn, Isai Thamizh, Winsley Rose, Baker Ninan Fenn, B. Gowrishankar, Narmada Ashok, Jacob John

**Affiliations:** 1 Department of Community Health, Christian Medical College, Vellore, 632002 India; 2 Department of Child Health, Christian Medical College, Vellore, India; 3 Department of General Medicine, Government Vellore Medical College and Hospital, Vellore, India; 4 Nalam Medical Centre and Hospital, Vellore, India

**Keywords:** Dengue, Vector-borne disease, South India, India, Dengue incidence, Dengue burden, Dengue surveillance, Dengue cost

## Abstract

**Background:**

Gaps in India’s routine dengue surveillance have led to an underestimation of its actual burden and could adversely affect funding for preventive public health measures. In this study, we report the incidence of symptomatic dengue and the associated direct medical costs in a large urban population-based cohort in Vellore, Tamil Nadu.

**Methods:**

Surveillance for febrile illnesses was conducted in 30 urban wards in Vellore as part of the Vellore Typhoid Vaccine Impact Trial. Participants seeking healthcare for febrile illnesses called the trial helpline, triggering active surveillance until fever defervescence. Febrile episodes lasting ≥ 3 days were diagnosed as dengue based on either laboratory tests or a physician-assigned diagnosis.

**Results:**

From May 2023 to January 2025, 74,587 participants aged 1–30 years contributed 124,018.4 person-years (PYs) of follow-up. We identified 188 laboratory-confirmed and 107 physician-assigned diagnoses of dengue. From May 2023 to April 2024, the incidence of laboratory-confirmed dengue was 150.3 per 100,000 PYs (95% confidence interval [CI]: 124.4–181.7), and 205.2 per 100,000 PYs (95% CI: 174.4–241.3) for all dengue cases. The median direct medical cost for laboratory-confirmed dengue treated as outpatients was Indian Rupees (INR) 4,357 (interquartile range [IQR]: 2,432–7,204), and INR 16,827 (IQR: 9,556–26,348) for those who were hospitalised.

**Conclusions:**

The incidence of dengue and its associated treatment costs were high in this urban setting. Strengthening routine disease surveillance with enhanced diagnostic testing and systematically collected data from the private sector will provide better estimates of the dengue burden in India and inform policy decisions.

## Background

The incidence of dengue has sharply increased over the past few decades. In 2024, the World Health Organization (WHO) reported a global outbreak of dengue fever, which affected over 14 million people and claimed the lives of more than 9,000 [1]. India mirrored this global trend, with reported cases increasing from approximately 28,000 in 2010 [2] to over 200,000 in 2024 [3], significantly contributing to the global dengue burden. This upward trend is expected to continue as global temperatures, international travel, and urbanization increase, potentially affecting millions of people and causing billions of dollars in economic damage [4].

Without antivirals available for the treatment of dengue [5], Indian policymakers might look to novel strategies, such as the *Wolbachia* method [6] and dengue vaccines [7, 8] for effective control. However, making informed decisions requires robust estimates of the disease burden and associated cost. Currently, the National Vector Borne Disease Control Programme (NVBDCP) and Integrated Disease Surveillance Programme conduct nationwide dengue surveillance. However, their figures may be underestimates, as evidenced by an analysis of the Global Burden of Disease study, which estimated 28.2 million dengue cases in India in 2021 [9], while the NVBDCP reported 193,245 cases for the same year [3]. Additionally, these programs do not account for the treatment costs incurred by patients, particularly those who use private healthcare. This study presents the incidence of symptomatic dengue and its direct treatment costs from a large, urban, population-based cohort (> 70,000 participants) in Vellore, Tamil Nadu, as part of the Vellore Typhoid Vaccine Impact Trial (VEVACT) [10].

## Methods

### Setting

This prospective community-based study included > 72,000 individuals aged 1–30 years residing in 30 urban wards of the Vellore Municipal Corporation, Tamil Nadu, India.

### Febrile surveillance

Acute febrile illnesses (≥ 38 °C) were recorded using a stimulated passive surveillance system established to monitor blood culture-confirmed typhoid cases, alongside an observer-blinded, cluster-randomized typhoid vaccine trial [10]. Participants contacted a study-associated helpline number in the event of a febrile illness. A field research assistant would follow up on the febrile episode daily, if the participant visited a healthcare facility, until they were fever free for 3 consecutive days. During these contacts, information was collected regarding the hospital visited, medicines administered, laboratory investigations, and physician-assigned diagnoses. The assignment of diagnoses and decisions to conduct laboratory tests were based on clinical algorithms followed by each hospital, which were not defined by VEVACT.

Dengue cases were classified as laboratory confirmed based on detection of non-structural (NS)-1 antigen or immunoglobulin (Ig) M antibody against the dengue virus by enzyme-linked immunosorbent assays (ELISA). In the absence of laboratory confirmation, physicians assigned a dengue diagnosis based on clinical findings and routinely available non-confirmatory tests. Only febrile cases that were medically attended and had a fever lasting at least 3 days were checked for dengue, as cases requiring medical intervention were expected to have a fever duration of at least 3 days [11]. The study offered reimbursement for medical expenses during a febrile episode from the date of the participant’s helpline call. Bills from the participants who sought reimbursement were collected and recorded by the study staff.

### Inclusion and exclusion criteria

All individuals aged 1–30 years at the time of recruitment to the VEVACT study who lived in the designated wards with no plans to leave for the next 24 months, were eligible for participation. Conversely, individuals with medical conditions that would prevent them from complying with the study requirements, such as follow-up and laboratory investigations, were excluded. All consenting individuals were included in the febrile surveillance.

### Public dengue surveillance data

We calculated the overall dengue incidence and case fatality rates (CFRs) in Tamil Nadu in 2024 using NVBDCP data [3]. Estimates of the state’s urban population in 2024 were obtained from India’s Census 2011 projections [12].

### Statistical analysis

Incidence, confidence interval (CIs), and person-time calculations were performed using Stata version 17 [13]. Odds ratios (ORs), means, medians, CFRs, and interquartile ranges (IQRs) were calculated using Stata version 17 (College Station, TX, USA). Graphs were created using ggplot2 [14] in R version 4.3.1 (Vienna, Austria) [15]. Follow-up time began on either May 1, 2023, or, if enrolment occurred afterward, on the date of consent. The follow-up ended on January 31, 2025, or the date of censorship, whichever came first. For incidence calculations, follow up time ended on April 30, 2024, to avoid seasonality bias. A 1-month period from the date of fever onset in dengue cases was considered a risk-free period in the analysis.

Overall, the dengue cases included physician-assigned and laboratory-confirmed cases. Alternative diagnoses were based on confirmatory tests or physician-assigned diagnoses. Hospitalized dengue cases were classified by severity according to WHO guidelines [16]. The incidence of dengue from routine public surveillance agencies was calculated as the total number of cases divided by the estimated urban population in Tamil Nadu for that year. Healthcare centers primarily used to treat febrile illnesses were categorized as public or private. Direct medical costs included physician consultations, pharmacy bills, laboratory investigations, and hospitalization expenses reported in Indian Rupees (INR). Costs reported in the cited studies were adjusted for inflation to 2024 using the inflation rates published by the Government of India [17].

## Results

From May 2023 to January 2025, 74,587 participants aged 1–30 years contributed 124,018.4 person-years (PYs) of follow-up time. The distribution of participants by age at recruitment and sex is presented in Table 1.

**Table 1:** Summary of the demographic details of study participants

Category	Participants (*n*)	% of Total
*Age group (years)*		
1–5	13,147	17.6
6–10	13,736	18.4
11–15	13,758	18.5
16–20	12,557	16.8
21–25	12,120	16.3
26–30	9,269	12.4
Total	74,587	100
*Sex*		
Male	36,858	49.4
Female	37,707	50.6
Other	22	0.03
Total	74,587	100

### Acute febrile illnesses

**Fig. 1:**
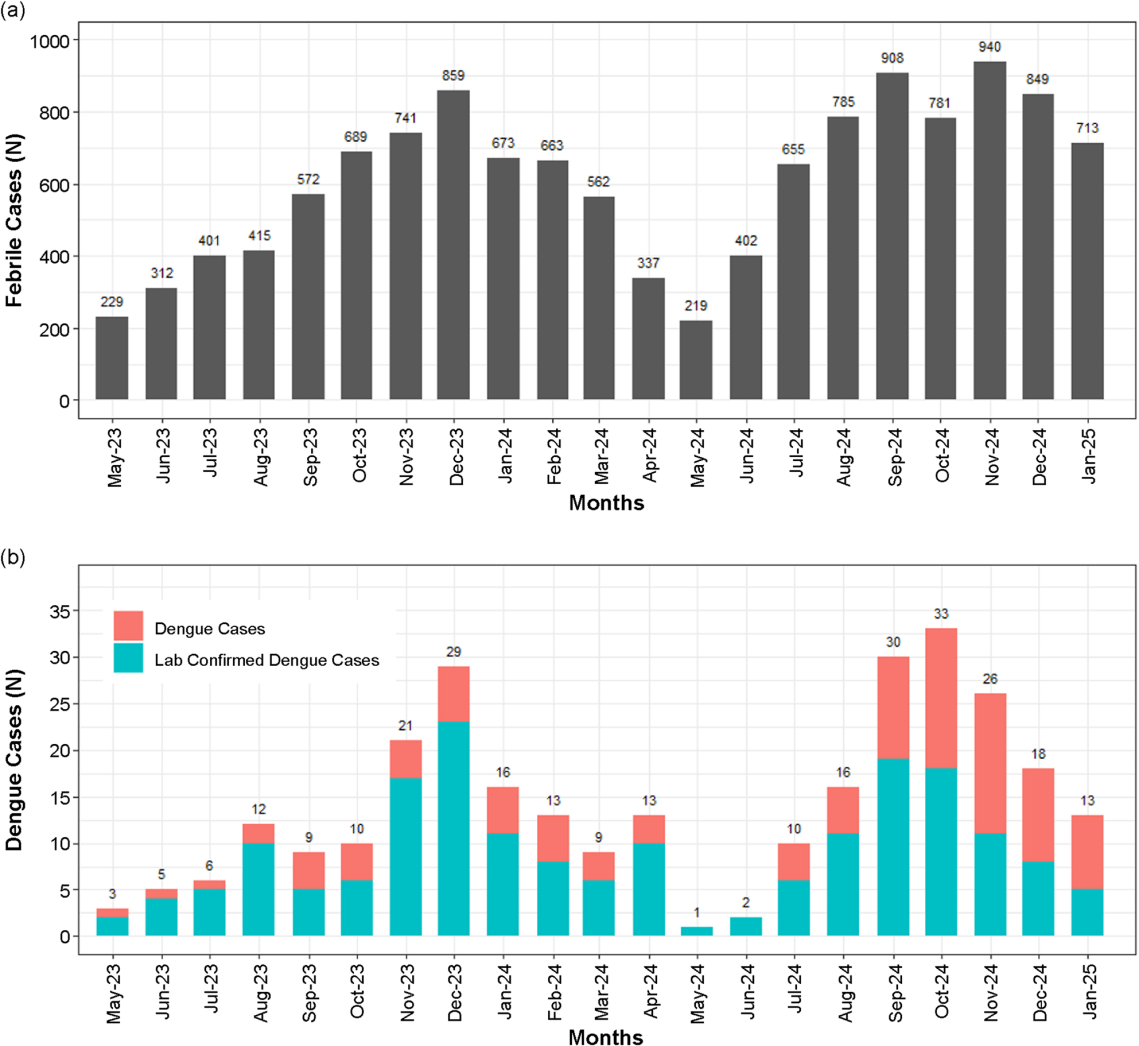
Month-wise distribution of medically attended febrile illnesses and dengue cases. (**a**) Month-wise distribution of medically attended febrile illnesses. (**b**) Month-wise distribution of dengue cases

During follow-up, 12,705 medically attended febrile illnesses (MAFIs) with fever lasting at least 3 days were recorded (Table 2), of which 188 cases (1.5%) were laboratory-confirmed dengue, and 107 (0.8%) were physician-assigned dengue. An alternative non-dengue diagnosis was observed in 4,724 (37.2%) of MAFIs, whereas 7,686 (60.5%) lacked an etiological diagnosis and were classified as acute febrile illnesses. Laboratory-confirmed dengue peaked in December 2023 and September 2024, whereas the overall dengue numbers were the highest in December 2023 and October 2024 (Fig. 1). The duration of illness and hospitalization details for dengue cases are presented in Table 3.

**Table 2:** Type of hospital (public or private sector) primarily used for treatment of febrile illnesses

Illness category	Overall *n* (%)*	Primary hospital	
		Public	Private
		*n* (%)*	*n* (%)*
*Outpatient*			
MAFIs^#^	11,460 (100)	3,114 (100)	8,346 (100)
Lab tested for dengue	185 (1.6)	14 (0.4)	171 (2.0)
Lab-confirmed dengue	40 (0.3)	1 (0.03)	39 (0.5)
All dengue	106 (0.9)	3 (0.1)	103 (1.2)
*Inpatient*			
MAFIs^#^	1,245 (100)	381 (100)	864 (100)
Lab tested for dengue	268 (21.5)	53 (13.9)	215 (24.9)
Lab-confirmed dengue	148 (11.9)	8 (2.1)	140 (16.2)
All dengue	189 (15.2)	18 (4.7)	171 (19.8)

#MAFIs- Medically attended febrile illnesses; *%- percentage of MAFIs

**Table 3:** Durations of fever and hospitalization (in days) of medically attended febrile illnesses (MAFIs)

MAFI category	*N*	Fever duration		Duration of hospitalization		Time from fever onset to hospitalization*		Time from discharge to defervescence#	
		Median	Range	Median	Range	Median	Range	Median	Range
*Outpatient*									
Non-dengue cases	11,354	4	3–34	-	-	-	-	-	-
Lab-confirmed dengue	40	5	3–16	-	-	-	-	-	-
All dengue cases	106	5	3–16	-	-	-	-	-	-
*Inpatient*									
Non-dengue cases	1,056	6	3–51	4	1–62	4	-6–22	0	-55–16
Lab-confirmed dengue	148	6	3–13	4	2–33	5	0–11	-1	-32–8
All dengue cases	189	6	3–14	4	1–33	5	0–11	-1	-32–8

*Negative values indicate hospitalization prior to fever onset; #negative values indicate defervescence prior to discharge

### Dengue incidence

From May 2023 to April 2024, the incidence of lab-confirmed dengue was 150.3 cases per 100,000 PYs (95% CI: 124.4–181.7), while the overall dengue incidence was 205.2 cases per 100,000 PYs (95% CI: 174.4–241.3). Distributed by age, the 6–10 years group had the highest incidence (Table 4) among both, lab-confirmed (242.6 per 100,000 PYs, 95% CI: 171.6–343.1) and overall (326 per 100,000 PYs, 95% CI: 241.8–439.6) dengue. Sex-specific incidence rates are listed in Table 4.

**Table 4:** Incidence of dengue within the study cohort per 100,000 person-years

Incidence Category	Total cases- *n*	Person-years	Incidence	95% CI
*Lab-confirmed dengue*				
By age group				
1–5	12	12,200.37	98.4	55.9-173.2
6–10	32	13,189.24	242.6	171.6-343.1
11–15	29	13,332.44	217.5	151.2–313
16–20	16	12,113.30	132.1	80.9-215.6
21–25	13	11,538.88	112.7	65.4–194
26–30	5	8,794.92	56.9	23.7-136.6
By sex				
Male	56	35,295.62	158.7	122.1-206.2
Female	51	35,851.53	142.3	108.1-187.2
Other	0	21.98	0	-
*All dengue cases*				
By age group				
1–5	22	12,199.55	180.3	118.7-273.9
6–10	43	13,188.34	326.0	241.8-439.6
11–15	36	13,331.86	270.0	194.8-374.4
16–20	20	12,113.02	165.1	106.5-255.9
21–25	19	11,538.38	164.7	105-258.2
26–30	6	8,794.84	68.2	30.6-151.9
By sex				
Male	76	35,293.98	215.3	172-269.6
Female	70	35,850.02	195.3	154.5-246.8
Other	0	21.98	0	-

### Dengue severity and case fatality

Among hospitalized laboratory-confirmed dengue cases (*n* = 148), 112 (75.7%) had one or more warning signs, 16 (10.8%) were severe, and five (3.4%) were severe and required intensive care for 2–5 days (range excludes one case where intensive care dates were not available). Of the 16 severe cases, two female individuals aged 11 and 21 years died during illness, yielding a CFR of 1.1% (95% CI: 0.1%–3.8%). Their causes of death were ‘dengue haemorrhagic fever with hypotensive shock’, and ‘dengue haemorrhagic fever with shock’, respectively.

Among the overall dengue cases that were hospitalized (*n* = 189), 137 (72.5%) had warning signs, 18 (9.5%) were severe, and 6 (3.2%) were severe and required intensive care. The overall CFR was 0.7% (95% CI: 0.1%–2.4%).

### Direct medical costs

Direct medical costs were available for 162 laboratory-confirmed dengue cases (Table 5). The median costs for those treated on an outpatient basis were INR 4,357 (IQR: 2,432–7,204) and INR 16,827 (IQR: 9,556–26,348) for those hospitalized. The median cost for severe dengue cases was INR 44,678 (IQR: 14,056–85,321), while for dengue with warning signs it was INR 17,685.5 (IQR: 10,875–24,735). The age group-specific costs are listed in Table 5.

**Table 5:** Direct medical costs (in Indian Rupees) for laboratory-confirmed dengue cases

Illness category	Outpatients		Inpatients	
	*N*	Median (IQR)	*N*	Median (IQR)
Overall	35	4,357 (2,432–7,204)	127	16,827 (9,556–26,348)
By hospital type*				
Private	35	4,357 (2,432–7,204)	125	16,846 (10,492–26,348)
Public	0	-	2	5,024 (3,605–6,443)
*By age group*				
< 15 years	27	4,239 (2,432–5,171)	81	14,800 (10,710–23,416)
≥ 15 years	8	8,457 (2,631–11,001)	46	21,990 (9,089–31,831)
*By dengue severity*				
Without warning signs	-	-	16	10,024 (6,007–16,702.5)
With warning signs	-	-	98	17,685.5 (10,875–24,735)
Severe dengue	-	-	13	44,678 (14,056–85,321)

*Type of hospital (public or private sector) primarily used for dengue treatment

### Healthcare utilization

MAFIs being treated at private healthcare facilities (Table 2) were more likely to receive dengue laboratory tests (OR: 2.23, 95% CI: 1.72–2.91, *p* < 0.001), be diagnosed with lab-confirmed dengue (OR: 7.68, 95% CI: 3.92–15.03, *p* < 0.001), and be classified as physician-assigned or lab-confirmed dengue cases (OR: 5.07, 95% CI: 3.25–7.93, *p* < 0.001) compared with those receiving care in public facilities.

## Discussion

Surveillance from the VEVACT cohort reported an incidence of 150.3 lab-confirmed dengue cases per 100,000 PYs (95% CI: 124.4–181.7) and 205.2 cases of laboratory-confirmed or physician-assigned dengue per 100,000 PYs (95% CI: 174.4–241.3). These rates significantly exceed those estimated using the NVBDCP data for Tamil Nadu in 2024 (66.4 per 100,000 PYs) and are comparable to regional rates in Southeast Asia (≥ 100 cases per 100,000 population) [18]. The highest incidence was among children aged 6–10 years, followed by those aged 11–15 years. This was consistent with previous findings [19] estimating that the force of infection for dengue in South India was highest in the 5–8-year-old group, followed by the 9–17- and 18–45-year-old groups. The CFR of 1.1% (95% CI: 0.13%–3.8%) among lab-confirmed dengue cases was similar to the reported national CFR (0.17%) [20] and higher than the calculated state-level CFR (0.05%). These results indicate higher baseline morbidity and mortality of symptomatic dengue in Vellore and possibly urban Tamil Nadu than previously expected from routine surveillance, stressing the need for effective control measures to be deployed within the city and state.

Routine surveillance likely underestimates dengue due to underreporting by the private healthcare sector and limited availability of diagnostics in the public sector, as other studies have shown [21, 22]. Further, we found that AFIs were more likely to be diagnosed with dengue (OR: 5.07, 95% CI: 3.25–7.93), have a lab-test for dengue (OR: 2.23, 95% CI: 1.72–2.91), and be diagnosed as a lab-confirmed dengue case (OR: 7.68, 95% CI: 3.92–15.03) when treated in private healthcare than the public sector. Our data, along with other studies [21, 22], suggest that improving reporting from private healthcare centers and diagnostics access in the public healthcare sector is needed to better estimate the true burden of dengue.

The median treatment cost for lab-confirmed outpatient cases in our study (INR 4,357) was similar to the 2016 estimate (INR 4,219.61; 1 USD = 66.26 INR in 2016, adjusted for inflation) [23]. Similarly, the median cost for hospitalized lab-confirmed dengue cases (INR 16,827) fell within the range of average hospitalization costs estimated for dengue in India by Ganeshkumar et al. in 2018 (INR 16,321.12–37,924.36; 1 USD = 63.80 INR in 2018, adjusted for inflation) [24].

However, hospitalization costs may have increased locally: among children aged < 15 years in our study, the median cost for hospitalized lab-confirmed dengue cases (INR 14,800) exceeded that reported in a smaller pediatric cohort from Vellore between 2016 and 2019 (INR 7,739.15, adjusted for inflation) [25].

These costs place a high economic burden on households. As a proportion of the mean per-capita expenditure (MPCE) for urban Tamil Nadu (INR 8,165) [26], the median direct cost for the treatment of outpatient lab-confirmed cases was 53.4% of the MPCE, which increased to 206% among lab-confirmed dengue cases that were hospitalized. These comparisons indicate that a significant proportion of households with members suffering from dengue may incur catastrophic health expenditure for treatment and experience financial distress.

Our study had some limitations. Some MAFIs seeking healthcare at non-study associated hospitals and clinics might not have been reported to the surveillance system which could have led to underestimation of dengue incidence. Owing to the nature of our surveillance, 60.5% of febrile cases had no etiological diagnosis, and febrile episodes that sought healthcare at pharmacies were not recorded, which potentially led to an underestimation of incidence in the cohort. Furthermore, we could only estimate the incidence and costs among individuals aged 18–30 years within the adult population, which reduced the generalizability of these estimates to older groups. Costs primarily reflect expenses in private healthcare facilities, as cost data were available for only two cases from the public sector. Finally, only costs for MAFIs that sought reimbursement were recorded. However, they were available for a majority (86.2%) of lab-confirmed dengue cases which should have reduced the associated bias.

Despite these limitations, our study identified a high incidence and treatment costs of symptomatic dengue among pediatric and young adult populations in Vellore, which may reflect the burden of dengue in urban Tamil Nadu. Additionally, there have been limited community-based studies on dengue in India. Our data should motivate similar research efforts nationwide to help establish a national understanding of the burden.

## Conclusions

We found a high incidence of dengue in Vellore despite a passive surveillance, and significant treatment costs. This indicates a need for intensified control efforts in the city. Further, our results suggest that strengthening India’s diagnostic laboratory network and systematic data collection from the private sector by routine disease surveillance agencies could improve estimates of the country’s dengue burden. Such measures would enable better informed policy decisions on control measures. This is especially important since several dengue vaccines are currently under trials in India and expected to become available in the coming years [27, 28]. 

## Data Availability

De-identified participant-level data will be made available via a public archive within three months of the primary VEVACT publication.

## Abbreviations

CFR: Case fatality rate
CI: Confidence interval
ELISA: Enzyme-linked immunosorbent assay
Ig: Immunoglobulin
INR: Indian Rupee
IQR: Inter-quartile range
IRB: Institutional review board
MAFI: Medically attended febrile illnesses
MPCE: Mean per-capita expenditure
NS: Non-structural
NVBDCP: National Vector Borne Disease Control Programme
OR: Odds ratio
PY: Person-year
VEVACT: Vellore Typhoid Vaccine Impact Trial
WHO: World Health Organization
